# Characterization of Aging-Associated Cardiac Diastolic Dysfunction

**DOI:** 10.1371/journal.pone.0097455

**Published:** 2014-05-28

**Authors:** Wei-Ting Chang, Jung-San Chen, Yung-Kung Hung, Wei-Chuan Tsai, Jer-Nan Juang, Ping-Yen Liu

**Affiliations:** 1 Division of Cardiology, Internal Medicine, National Cheng Kung University Hospital, Tainan, Taiwan; 2 Institute of Clinical Medicine, National Cheng Kung University, Tainan, Taiwan; 3 Department of Engineering Science, National Cheng Kung University, Tainan, Taiwan; Texas A & M, Division of Cardiology, United States of America

## Abstract

**Aims:**

Diastolic dysfunction is common in geriatric heart failure. A reliable parameter to predict myocardium stiffness and relaxation under similar end-diastolic pressure is being developed. We propose a material and mathematical model for calculating myocardium stiffness based on the concept of linear correlation between 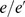 and wedge pressure.

**Methods and Results:**

We enrolled 919 patients (male: 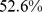 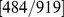). Compared with the younger population of controls (mean age: 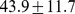 years; 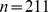; male: 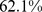 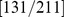), the elderly (mean age: 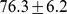; 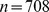; male: 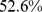 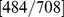) had a greater prevalence of hypertension, diabetes mellitus, and coronary artery disease (all 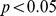). We collected their M-mode and 2-D echocardiographic volumetric parameters, intraventricular filling pressure, and speckle tracking images to establish a mathematical model. The feasibility of this model was validated. The average early diastolic velocity of the mitral annulus assessed using tissue Doppler imaging was significantly attenuated in the elderly (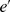: 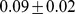 vs. 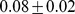; 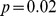) and corresponded to the higher estimated wedge (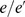) pressure (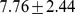 vs. 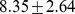; 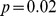) in that cohort. E (Young's modulus) was calculated to describe the tensile elasticity of the myocardium. With the same intraventricular filling pressure, E was significantly higher in the elderly, especially those with 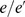 values 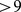. Compared with diastolic dysfunction parameters, E also presented sentinel characteristics more sensitive for detecting early myocardial relaxation impairment, which indicates stiffer myocardium in aging hearts.

**Conclusion:**

Our material and geometric mathematical model successfully described the stiffer myocardium in aging hearts with higher intraventricular pressure. Additional studies that compare individual differences, especially in health status, are needed to validate its application for detecting diastolic heart failure.

## Introduction

Heart failure is increasingly prevalent among older adults [1]. Clinically,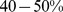 of patients with symptomatic heart failure have preserved left heart function, with preserved ventricular ejection fraction (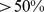) [2]. Sometimes this is called “diastolic dysfunction with preserved systolic function” or “diastolic heart failure” [3]. People with diastolic heart failure are generally older and female, and tend to have a greater incidence of systemic hypertension than do those with contractile dysfunction (“systolic heart failure”) [4], [5]. Physiologically, diastolic heart failure occurs when the ventricle cannot fill properly because it cannot relax or its wall is too rigid [6]. Histological evidence supports the notion that diastolic dysfunction is related to ventricular hypertrophy, increased interstitial collagen deposition into the myocardium [7]. Similarly, aging hearts continuously lose myocytes, which is compensated for by reactive hypertrophy of the remaining cells; thus, these hearts are filled with fibrotic or adipose tissue [8]. Histological samples for measuring the exact tensile elasticity of the myocardium are possible only in animal studies, not human studies. Some mathematical models have been used to describe the dynamics of remodeling in skeletal muscle, arteries, and even the heart [9], [10]. However, a mathematical model to study cardiac aging is still lacking. Establishing a model of the aging heart would provide a tool for us to understand underlying mechanisms of cardiac aging and to define the impaired myocardial relaxation process.

It remains uncertain whether current available invasive and noninvasive diagnostic tools can accurately predict myocardium stiffness. The time constant of relaxation (tau, 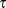), which describes the rate of left ventricular (LV) pressure decay during isovolumic relaxation, is currently the standard parameter for predicting the relaxation function of myocardium [11], [12]. However, several confounding factors, when echocardiography is used to measure the deceleration time of mitral inflow, may disturb the equivalence between echocardiographic and catheterized results. Despite the ratio of mitral inflow to annulus tissue, Doppler imaging velocity (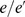) indicates the intraventricular pressure, which is within the borderline range of elevated pressure (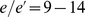), the discrimination of diastolic dysfunction remains a dilemma [3]. Therefore, a reliable and noninvasive diagnostic parameter is crucial for facilitating an accurate diagnosis that indicates whether the stiffness is myocardial stiffness.

Studies [13]–[21] on the elastic properties of the contracting left ventricle have aroused a great deal of interest among scientists and engineers. Many researchers have developed a series of experimental techniques for determining the elastic properties of the left ventricle. A simple and practical approach for in vivo determinations of the properties of the canine left ventricle, proposed in 1972 [13], established the relationship between the effective elastic modulus and the circumferential stress throughout the isovolumetric systolic period. After the concept on the elastic properties of the left ventricle was accepted, some researchers reported that the effective modulus E measured from experiments could also be viewed as an additional indication of the left ventricle having adjusted to the heart disease [14], which showed that normal values of E during the systole directly indicate that the strength of the left ventricle contraction is normal. The nomogram, a clinically usable closed-chest procedure for determining the elastic modulus, was introduced in 1975 [15]. Using a heart-sound-frequency analysis followed by a determination of E, the loss of muscle-medium elasticity can be roughly delineated. In addition to improving data-acquisition techniques, the modeling of the left ventricle is becoming a more important factor for determining myocardial elasticity. These findings showed that the stiffness of the complete ventricle should be considered a function not only of myocardial stiffness but also of the cavity shape, dimensions, and structure of the vessel [16]. A better approximation for ventricular modeling requires assuming that myocardial stiffness is a function of geometry and stress. In other words, the geometry of the left ventricle is essential for simulating it in a model. Thick-walled models are commonly and widely used to study the dynamics of the ventricle. Several studies [17]–[19] on cardiac muscle mechanics, LV pump function, and LV wall thickening view the left ventricle as a thick-walled cylindrical composite. They report that the thick-walled cylindrical framework seems to be a good and practical approximation sufficient for simulating the left ventricle. One study [20] presented a simple analytical model to describe the relationship between age-related changes in the structure and function of mouse cardiac muscle. It suggested that age-related cardiac sarcopenia likely contributes to depressed LV function in the absence of overt cardiovascular disease. Recently, an alternative mathematical model for investigating cardiac aging characterized by diastolic dysfunction of the left ventricle was introduced [21]. In contrast to the previous study, a spherical thick-walled model and stretch-induced tissue-growth postulate were used to predict LV dimension and wall stiffness changes in aging mice. The Young's modulus of the left ventricle was determined by introducing a smooth monotonic function to fit the experimental data and a simplified version of the linear mixture theory of composite material. It was assumed that the pressure difference and the Young's modulus of the left ventricle are two independent factors that affect end-diastolic dimension/diameter and wall thickness.

The importance of the aging effect on the large vessels and cardiac structure can be also seen from a study [22] on the effect of hypertension on the diameter and elastic modulus of the aortic arch; it showed that the elastic modulus was significantly correlated with age in patients with, but not without, hypertension. In addition, both the aortic arch diameter and the elastic modulus are larger in patients with sustained uncomplicated essential hypertension. Based on the proven linear correlation between mitral e velocity, corrected for the influence of relaxation (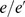 ratio) and intraventricular pressure (similar to wedge pressure; 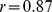; pulmonary capillary wedge pressure (PCWP)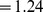 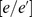 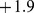) [23], we have created a cardiac mathematical model to simulate the remodeling process under various pressures during the aging process. Unlike other research groups, we hypothesized that the pressure, elastic modulus, and LV dimension are mutually influenced, that the relationship of the elastic modulus to pressure, wall thickness, and age can be established with the mathematical model, and that the aging effect on LV wall thickness can also be determined using the fixed pressure.

## Materials and Methods

### Patients

We enrolled 919 patients (male: 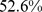 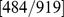) and divided them into two cohorts: (1)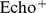: patients given echocardiography on a physical examination at our university hospital between February 2012 and June 2013, and (2) TOP (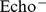): the Tianliao Old People (TOP) study between July 2010 and August 2012 [24], [25]. Echocardiographic parameters based on the recommendations of the American Society of Echocardiography [26], medical records, and clinical questionnaires were collected from patients in the cohort. Patients with a poor image window, LV systolic dysfunction, or significant (>moderate severity) valvular heart disease were excluded. This study was approved by the Institutional Review Board of National Cheng Kung University Hospital (IRB no: ER-99-111), and each patient signed an informed consent form before the physical examination.

### Echocardiography

**Figure 1:**
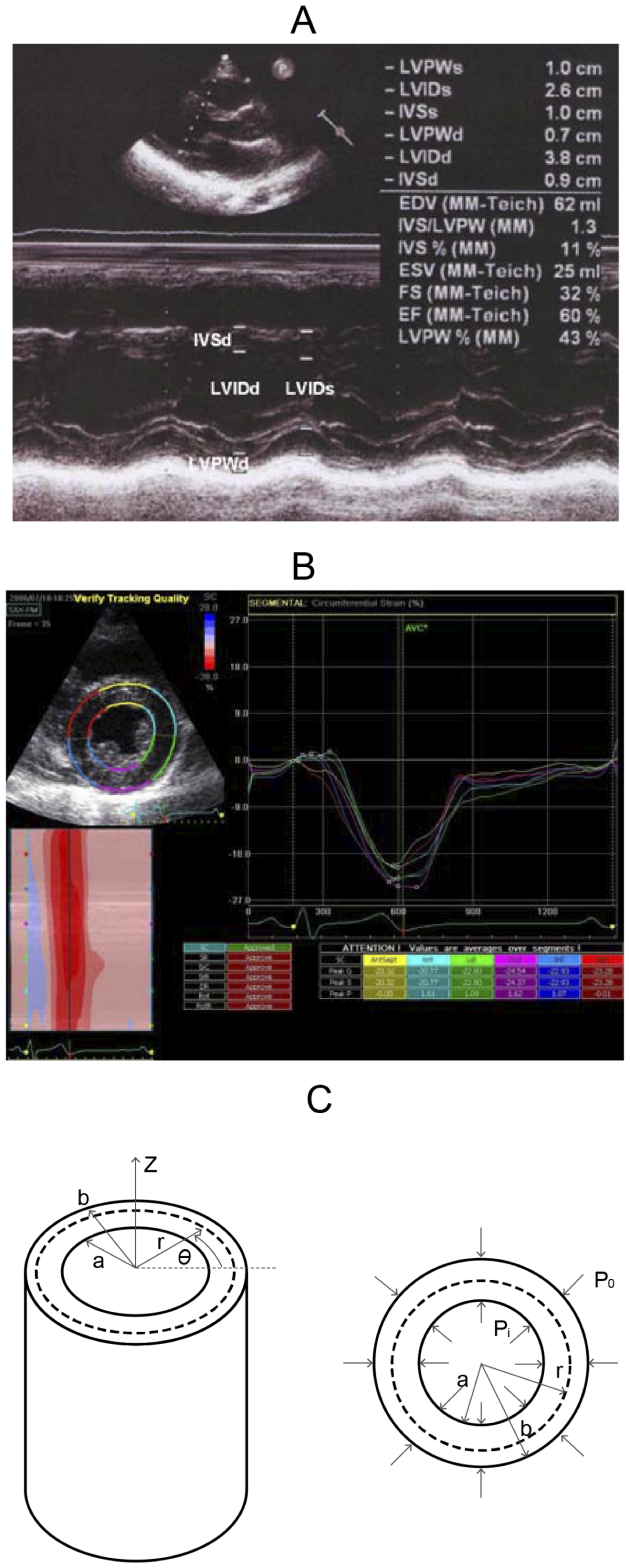
The echocardiographic parameters and the mathematical model for computing the myocardium stiffness of the left ventricle. A: The measured echocardiographic parameters. IVSd: intraventricular septal width in diastole; LVIDd: left ventricular internal dimension in diastole; LVPWd: left ventricular posterior wall width in diastole; and LVIDs: left ventricular internal dimension in systole. B: The measuring circumferential strain in the short-axis, at the papillary muscle level. C: The model of the cylinder, gross and cross-section.

Standard echocardiography was done (Vivid I; GE Vingmed Ultrasound AS, Horten, Norway) using a 3.5-MHz multiphase-array probe. The chamber dimensions and LV mass were measured using the two-dimensionally guided M-mode method, and the LV ejection fraction (LVEF) was measured with the two-dimensional M mode (Figure 1A). Intraventricular septal width (IVSd), LV internal dimension (LVIDd), LV posterior wall width (LVPWd), and LV internal dimension (LVIDs) were measured sequentially to calculate geometry and ejection fraction. Transmitral Doppler flow velocity was obtained from an apical four-chamber view, and peak early filling velocity (e), peak atrial velocity (a), and the 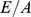 ratio were recorded. Early diastolic annular velocity (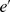) and atrial annular velocity (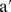) were also measured to estimate the LV end-diastolic pressure (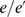). The average of medial and lateral 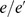 was used to represent the estimated intraventricular pressure.

To measure the circumferential strain, 20 patients were randomly selected from the 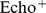
cohort to receive speckle-tracking echocardiography (STE) (Figure 1B). Short-axis views at the papillary muscle level were recorded in digital loops for a deformation analysis of the left ventricle. The images were acquired at 70–90 frame/sec and stored for three cycles. The images were analyzed offline using computer software (EchoPAC 09; GE-Vingmed Ultrasound AS, Horten, Norway). After tracking the margin of endocardium, the software detected the myocardial motion during the entire cardiac cycle. The circumferential strain of six segments was averaged and used in the computation for the condition that 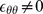.

### Statistical analysis

SPSS 18.0 (SPSS Inc., Chicago, IL) was used for data management and statistical analyses. Data are means ± standard deviation (SD). Continuous variables were compared using Student's *t* test for normally distributed values. Significant factors in univariate analysis were entered into multivariate analysis. Multivariate logistic regression analysis was used to identify the independent significance of E in patients with diastolic dysfunction. A Pearson's partial correlation coefficient (*r*) between E and 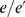 was calculated. Statistical tests were 2-sided; significance was set at 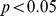.

### Mathematical modeling

In this section, a mathematical model for computing the myocardium stiffness of the left ventricle is presented. The left ventricle is assumed to be made of elastic, isotropic, and homogeneous tissue that will completely recover its native form when the forces are removed. To capture LV wall dynamics, a thick-walled cylindrical pressure vessel was used (Figure 1C). The cylinder allows reasonably complex motions of the left ventricle, viz., radial inflation, axial extension, torsion, and transmural shear reflecting a cross-sectional view of the cylinder. The uniform internal (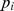) and external (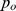) pressures are respectively applied to the inner and outer surfaces of the cylinder. The interior radius and exterior radius of the cylinder are denoted by *a* and *b*, respectively. The quantity *r* denotes the radius at an arbitrary position between *a* and *b* [27].

For simplification, the strain normal to the cross-sectional plane (*r*−*θ* plane), 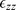, and the shear strains 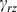 and 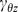 are assumed to be zero. Hence, the present three-dimensional problem can be reduced to an equivalent two-dimensional one involving approximation. In addition, the cylinder is assumed to be axisymmetric, i.e., the deformation and loading conditions of the cylinder are independent of 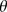. Then the radial stress 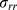 and circumferential stress 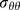 can be readily obtained as
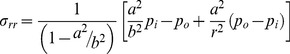
(1)

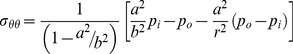
(2)The corresponding strains can be acquired from the constitutive relation (stress-strain relation), namely
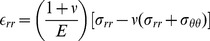
(3)

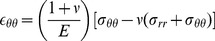
(4)where 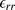 is the radial strain, 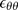 is the circumferential strain, 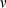 is Poisson's ratio, and E is the Young's modulus. Solving Eq. (3) and Eq. (4) yields
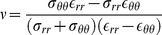
(5)

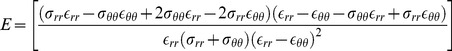
(6)Substituting Eqs. (1) and (2) into Eq. (6) gives
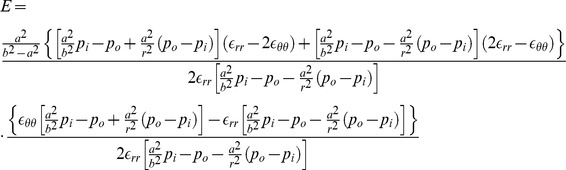
(7)


If 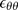 is not considered, Eqs. (5) and (6) can be reduced to
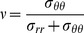
(8)

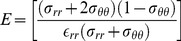
(9)Then the Young's modulus for 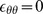 can be readily derived as
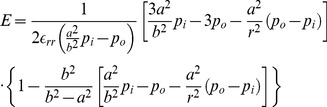
(10)The parameters *a*, *b*, 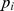, and 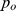 can be acquired from experiments and shown as follows:
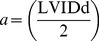
(11)

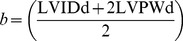
(12)

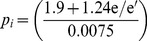
(13)

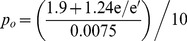
(14)Also, 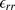 is the ratio of the radial elongation to the diastolic radius, i.e., 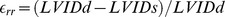, and 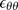 is an average strain for six segments in the circumferential direction. For computation, the parameter *r* in Eqs. (7) and (10) was chosen as 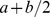.

## Results

### The clinical and echocardiographic characteristics of the younger and older cohorts

The elderly cohort (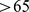 years old) were significantly older (mean age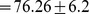 vs. 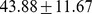 years), had a higher prevalence of hypertension (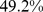 vs. 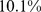), diabetes mellitus (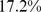 vs. 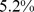), and coronary artery disease (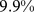 vs. 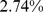; all 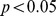) (Table 1) than did the younger cohort (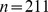; male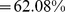). All of them worked and lived independently. Based on our previous questionnaire in the TOP study [25], most lived an active life (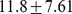 h of walking/week).

**Table 1:** The clinical and echocardiographic characteristics of the younger and older cohorts.

Variable	Younger cohort n = 211 (22.9%)	Older cohort n = 708 (77.1%)	p-value
Age (years)	43.88±11.67	76.26±6.2	<0.001
Male	131 (62.08)	484 (68.82)	<0.001
HTN	21 (9.95)	349 (49.22)	<0.001
DM	11 (5.21)	122 (17.2)	<0.001
CAD	6 (2.84)	71 (10.01)	0.02
Echocardiographic parameters			
IVSd (cm)	0.81±0.2	0.78±0.2	0.14
LVPWd (cm)	0.73±0.1	1.20±6.6	0.31
LVIDd (cm)	4.78±0.5	5.03±0.6	0.01
LVIDs (cm)	2.90±0.4	3.05±0.5	0.01
LVEF (%)	70.02±6.7	68.20±8.4	0.09
e (m/s)	0.74±0.2	0.65±0.3	0.01
e/a	0.80±0.8	0.70±0.3	0.14
*e′* (m/s)	0.09±0.02	0.08±0.02	0.02
*e/e′*	7.76±2.44	8.35±2.64	0.02
IVRT	94.44±21.25	107.77±19.1	0.09
DT	210.91±69.52	179.01±94.66	0.67
E (Young's modulus)	28872.72±7710.74	31325.97±10275.77	0.001

Data are 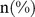 or mean or 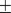 standard error; HTN = hypertension; DM = diabetes mellitus; CAD = coronary artery disease; IVSd = inter-ventricular septal diameter in diastolic phase; LVPWd = left ventricular posterior wall diameter in diastolic phase; LVIDd = left ventricular internal diastolic dimension; LVIDs = left ventricular internal systolic dimension; e = early diastolic mitral inflow velocity; e/a = the ratio of early to late diastolic mitral inflow velocity; e′ = the average early diastolic velocity of mitral annulus in tissue Doppler; LVEF = left ventricular ejection fraction.

To establish a model for predicting the stiffness characteristics of myocardium, we used the M-mode and 2-D echocardiographic volumetric parameters, intraventricular filling pressure, and STE from all clinical patients and the 2 cohorts. The feasibility of this model was validated. Despite a slight but significant (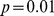) difference in intraventricular diameter between the two cohorts (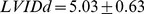 vs. 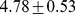), the LV systolic ejection fraction was within the normal range in all patients (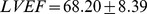 vs. 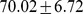; 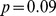). The mean early diastolic velocity of the mitral annulus in tissue Doppler imaging was significantly attenuated in the elderly cohort (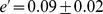 vs.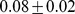; 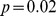), which corresponds to the higher estimated wedge (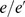) pressure (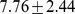 vs. 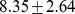; 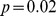). E (Young's modulus) was calculated to describe the tensile elasticity of the myocardium, which was also significantly (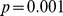) elevated in the elderly cohort (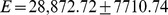 vs. 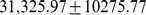). Instead of a linear relationship, the regression analysis illustrated a nonlinear association between age and E (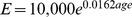; 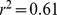)

After excluding patients with hypertension, diabetes mellitus, and coronary artery disease, the correlation between E and diastolic dysfunction remained significant (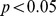) in the healthy elderly compared with other echocardiographic parameters (Table S1).

### The relationship between E and echocardiographic intraventricular pressure in the younger and older cohorts

**Figure 2:**
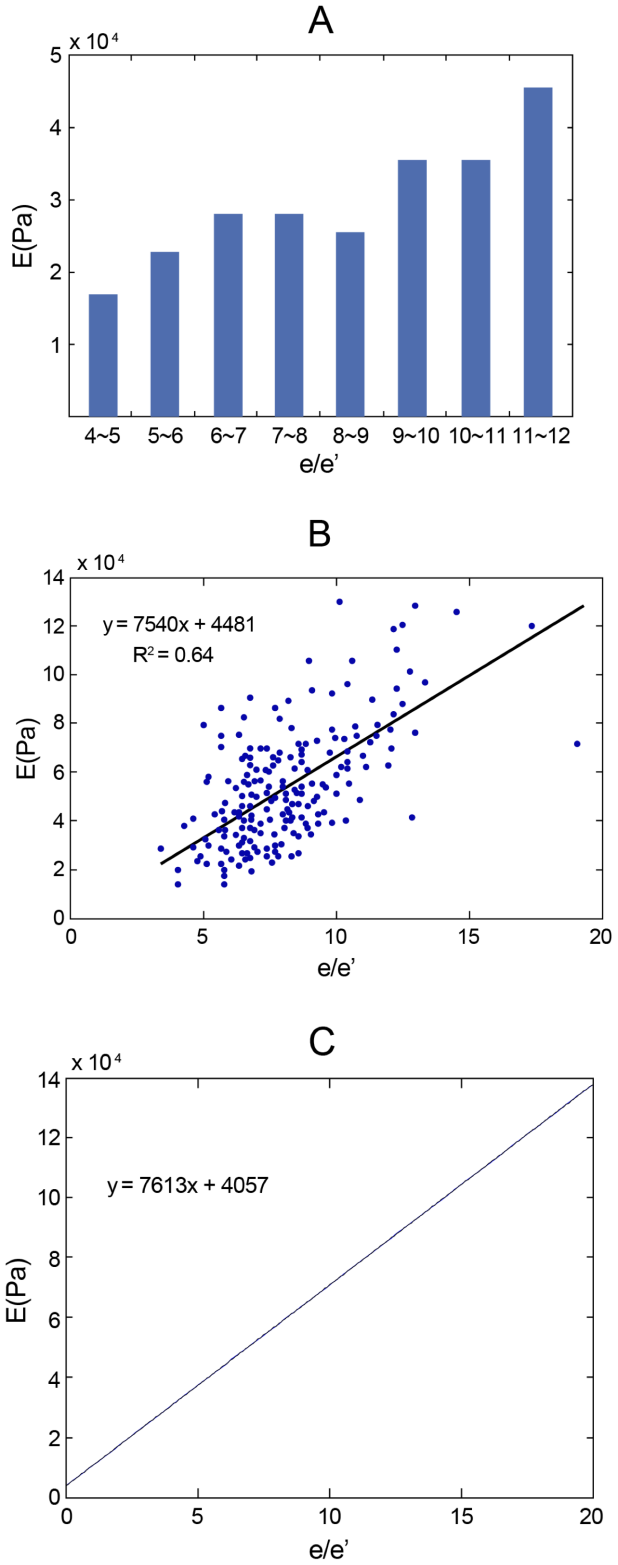
The correlations between 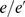 and E both in the clinical statistics and the mathematical models. A and B: The relationship between 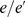 and E in clinical statistics, and C: in the mathematical model. 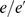 = estimated intraventricular pressure by the ratio of early diastolic mitral inflow velocity and the averaged early diastolic velocity of mitral annulus in tissue Doppler imaging; 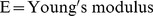.

**Figure 3:**
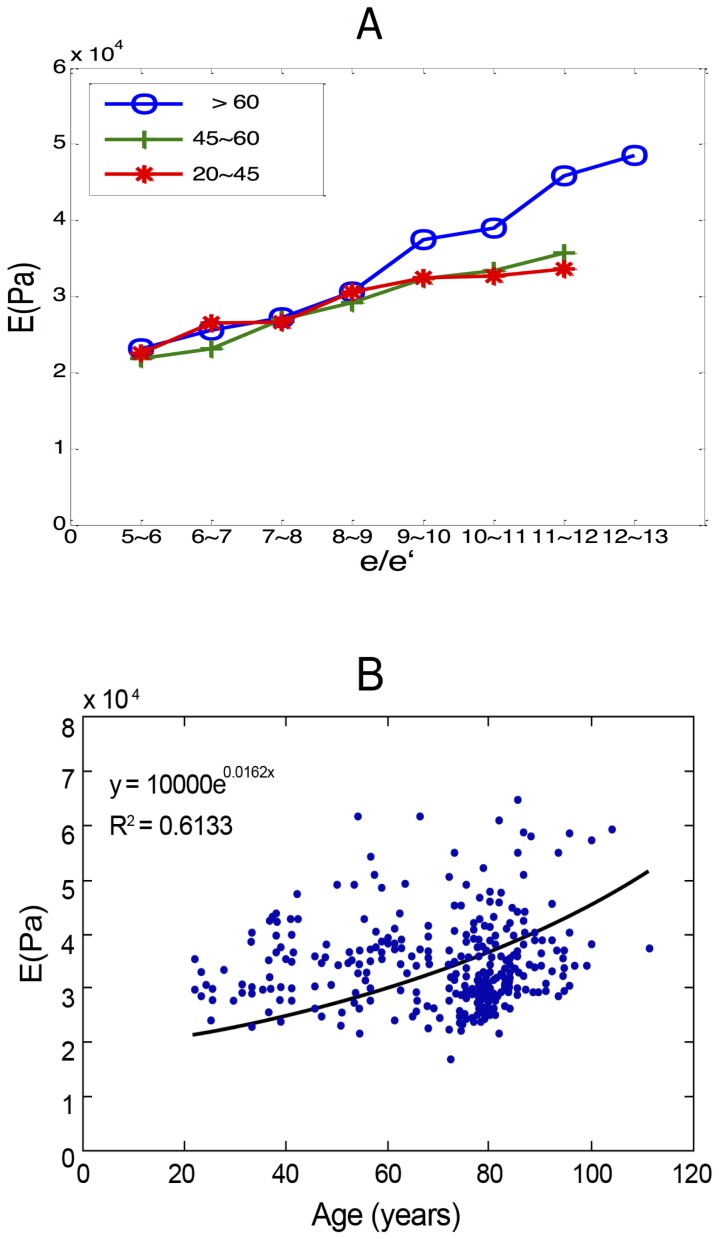
The relationship between E and echocardiographic intraventricular pressure in the younger and older cohorts. A: The relationship between 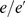 and E value in various age group. B: The exponential correlation between age and E in the regression analysis.

There was a positive correlation between 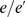 and E (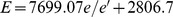; 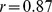; 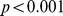) both in the clinical statistics (Figure 2A,B) and in the mathematical models (Figure 2C). The slope of the trend line shown in Figure 2B is very close to the one obtained by directly using the mathematical model in Figure 2C. In the group with an 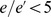, E was estimated as 18,042(Pa), but once the pressure elevated (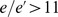), E significantly increased (45,030 Pa). Categorizing patients by age groups (20–45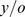, 45–60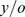, 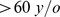) showed that, although under the same intraventricular filling pressure, E was significantly higher in the elderly (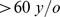), especially with a higher 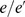 (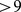) (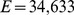 in 20–45-year-old group, 33,778 in 45–60-year-old group, and 42,726 in >60-year-old group) (Figure 3A). However, the difference was not significant in younger patients or between genders. Regression analysis showed that an exponential correlation between age and E could be summarized to delineate the soaring E in older patients (Figure 3B). Multivariate analysis, compared with other traditional parameters for diagnosing diastolic dysfunction (deceleration time [DT]; isovolumic relaxation time [IVRT]), E also showed independent elevation for detecting early myocardial relaxation impairment in both younger (odds ratio 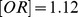; 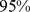 confidence interval 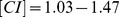; 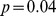) and older patients (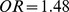; 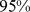 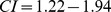; 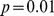), which indicates stiffer myocardium in aging hearts. (Table 2 and 3; Table S2 and S3)

**Table 2:** Multivariate regression analyses for the independence of E value in younger patients with diastolic dysfunction.

Variable	Odds Ratio	95% Confidence Interval	p-value
Age	1.33	0.66–2.68	0.41
Diabetes Mellitus	0.46	0.18–1.13	0.09
IVSd	1.02	0.46–2.23	0.96
IVRT	0.99	0.93–1.02	0.11
E (Young's modulus)	1.12	1.03–1.47	0.04

Abbreviations: see Table 1.

**Table 3:** Multivariate regression analyses for the independence of E value in older patients with diastolic dysfunction.

Variable	Odds Ratio	95% Confidence Interval	p-value
Coronary Artery Disease	1.01	0.97–1.03	0.62
e/a	0.46	0.18–1.13	0.90
E (Young's modulus)	1.48	1.22–1.94	0.01

Abbreviations: see Table 1.

### The corresponding trend in various models

**Figure 4:**
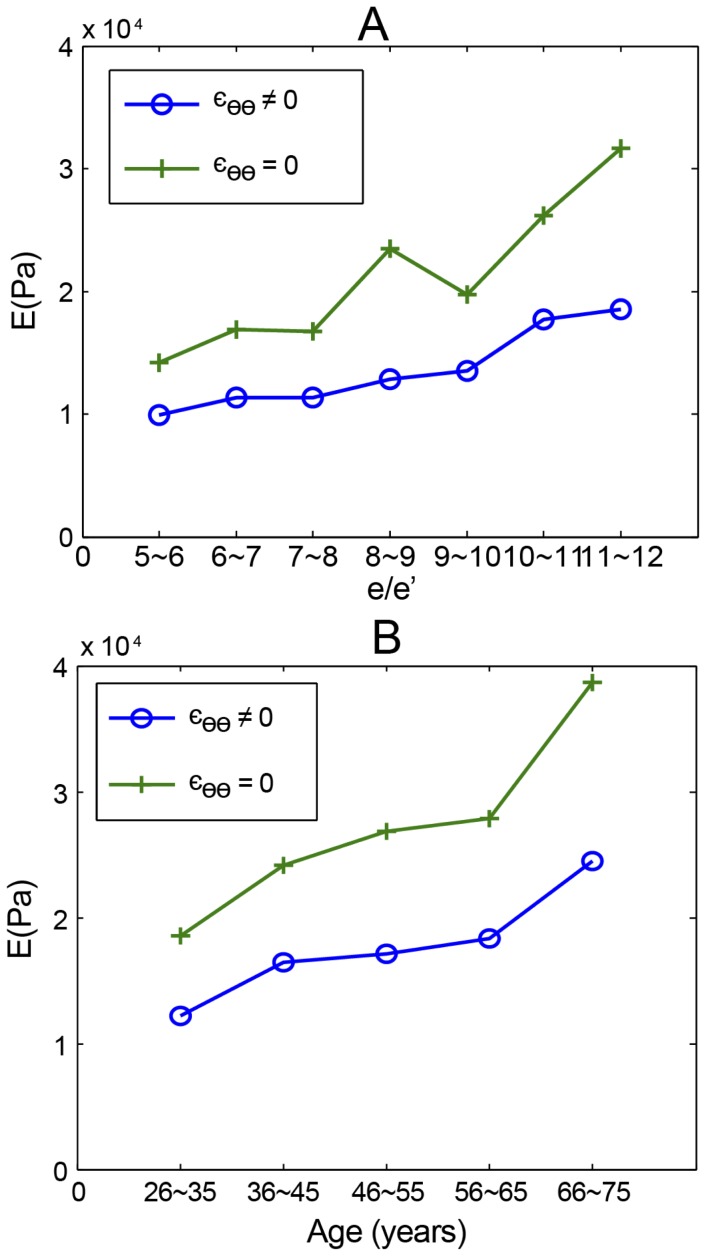
Various models to simulate actual hearts with different conditions. A: The positive association between 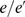 and E, and B: between age and E in the mathematical model, whether or not considering circumferential strain. 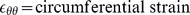.

To simulate the human heart, we created a model to obtain the circumferential strain. In addition to the radial strain, the circumferential strain of the actual myocardial contraction was measured using STE Besides a grossly attenuated E, the model reflected a similar trend of geometric and pressure change, regardless of whether the circumferential strain was considered (Figure 4 A,B). Thus, if the major question is the relationship between pressure and the tensile elasticity of the myocardium, rather than the exact values of E, the simpler cylinder model may replace the complex elliptical model to reduce measurement and calculation errors.

### The relationship between E and echocardiographic wall thickness in the younger and older cohorts

**Figure 5:**
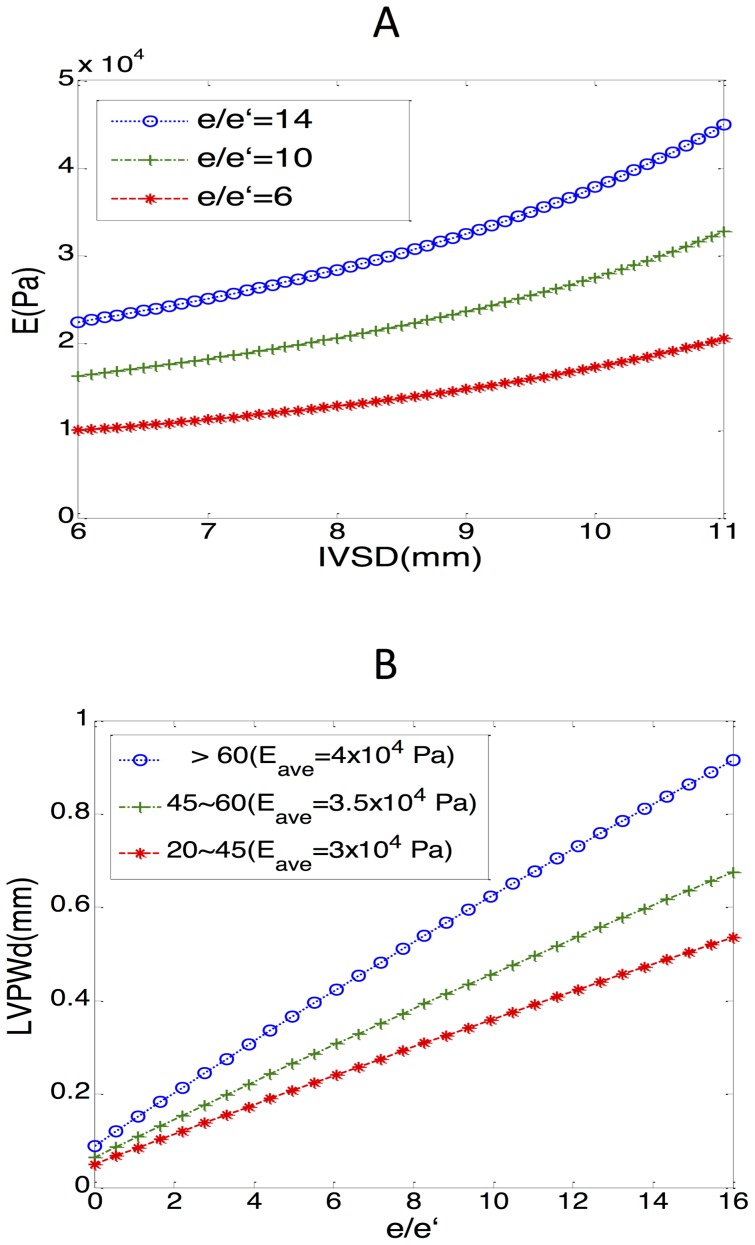
The relationship between E and echocardiographic ventricular wall thickness in the younger and older cohorts. A: In the mathematical model, the positive association between IVSd and E when the value of 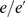 is low, moderate, and high. B: The positive correlation between 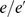 and IVSd with different average values of E (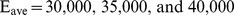 for the 20–45, 45–60, and 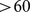 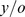 age groups, respectively). Because circumferential strain = 0, a (internal dimension), pressure, E = f (LVPWd), or E = f (IVSd) was fixed. IVSd = intraventricular septal width in diastole; LVPWd = left ventricular posterior wall width in diastole.

LV hypertrophy was more prevalent in the elderly than in the younger and middle-aged patients; it led to poor compliance and to difficulty in shape changing; thus, it impaired diastolic function. Although the association between a thicker wall and higher intraventricular pressure has been frequently reported, a suitable model to illustrate it in patients of various ages is lacking. Our mathematical model depicted positive associations between IVSd and E, along with the increasing 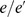 (Figure 5A). It also indicated that in the same LV wall width, a higher intraventricular pressure correlated to a higher value of E.

In addition, by inputting the representative E (30,000, 35,000, and 40,000 for the 20–45, 45–60, and 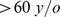 groups, respectively), we discovered that a higher value of E, along with aging, was associated with a thicker ventricular wall under the same intraventricular pressure (Figure 5B). This may explain the deteriorating myocardial relaxation and higher incidence of signs of heart failure signs in the elderly, who are more vulnerable to the same pressure.

## Discussion

This is the first evaluation of the diastolic dysfunction in the aging human heart that uses physiologic and echocardiographic assessments combined with mathematical modeling. Our most important findings are that: (1) E (Young's modulus), the tensile elasticity of the myocardium, can be used clinically to describe myocardial relaxation and to noninvasively detect early diastolic dysfunction; (2) aging myocardium becomes thicker, stiffer, and less expandable, which agrees with previous clinical findings. Even under the same intraventricular filling pressure, E was significantly higher in elderly patients (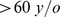), especially with a higher 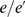 (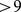); (3) our mathematical model similar trends of geometric and pressure changes, regardless of whether circumferential strain was considered.

The criteria for diagnosing diastolic dysfunction and diastolic heart failure remain imprecise [3], [11]. Although there are several parameters that indicate myocardial relaxation, none directly describe the tensile property of myocardium. In addition to some measurement errors, the current diagnostic criteria are nsufficient when the borderline value of estimated intraventricular pressure (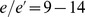) is considered [12]. Another dilemma is the coexistence of systolic and diastolic dysfunction [27]. Different from diastolic dysfunction, which remains preserved in myocardial structure and contractions, systolic dysfunction leads to fatigue and a failure of adequate deformation. Because echocardiographic parameters are too limited to precisely delineate diastolic function in systolic heart failure, using E offers the physician a new opportunity to distinguish it from other types of heart failure based on the change in myocardial stiffness [28].

Researchers have discovered a continuous loss of myocytes surrounded by the adipose and fibrotic tissue deposited in the extracellular matrix (ECM) in the aging process [7]. Therefore, myocardial stiffness is determined by the volume ratio and the combined mechanical properties of the myocytes and ECM. To prove that the age-related increase of collagen content shifts myocardial mechanical properties from a myocyte-based stiffness to a collagen-based stiffness, researchers have created a number of mathematical models [13]–[21]. To describe myocardial stiffness in various stages, the histological components of myocytes and ECM were transformed to different input numbers in those models. Yang et al. [21] created a mathematical model of LV remodeling in aging mice. Because human myocardial samples are insufficient to support similar models of the aging human heart, we replaced the histological parameters with clinical data. Other studies [22]–[23] have reported that the 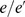 ratios is highly correlated with intraventricular pressure, enabled us to illustrate the geometric and tensile changes under different pressures.

In Yang et al. [21], a spherical thick-walled model and stretch-induced tissue-growth postulate were used to predict left ventricle dimensions and wall stiffness changes in aging mice. A generalized Hook's Law was considered and used for calculating the strain in the radial direction. The Young's modulus of the left ventricle was determined by introducing a smooth monotonic function to fit the experimental data and a simplified version of the linear mixture theory of composite material. It was assumed that the modulus depends only on the volume fraction of collagen and the Young's moduli of collagen and muscle. Also, the pressure difference and the Young's modulus of the left ventricle were assumed to be two independent factors affecting wall thickness and end-diastolic dimension and diameter. The changes in left ventricular mass and pressure with time are important throughout the whole study. It was concluded that senescent mice tend to have a higher modulus and pressure than do young mice.

In our study, the left ventricle considered as a pressurized thick-walled cylinder is assumed to be made of one type of elastic, isotropic, and homogeneous tissue that will be completely restored its native form when the forces are removed. We restricted the equations (stress-strain relations) to the case of plane elasticity. Strains in the radial direction and in the circumferential direction can be taken into account when determining the stiffness of myocardium. The radial and circumferential strains measured from experiments were directly used to calculate the Young's modulus (E), which is currently regarded as a practical indicator for evaluating cardiac diastolic dysfunction. Also, in our mathematical model, the pressure, elastic modulus, and left ventricle dimensions are mutually influenced. We found that when using the same intraventricular filling pressure to compare the elderly and younger patients, the former had a tendency to have a higher E value. (Table 4) Because data-fitting techniques were not used in our computation, more accurate results could be obtained. In summary, our mathematical model offers an additional practical approach to evaluate cardiac diastolic dysfunction.

**Table 4:** Comparison of our model and the previous Yang et al. model.

	Yang et al. model [21]	Our model
Geometric assumption	Sphere	Cylinder
Stress-strain relation	Hook's law	Plane elasticity
Material	Composite material	Isotropic material
Factors influencing Young's modulus	Young moduli of collagen	Interior and exterior pressure (measured from experiment)
	Young moduli of muscle	Initial dimension (measured from experiment)
	Volume fraction of collagen (data fitting)	Strains (measured from experiment)

STE is an emerging technique, with angle- and load-independent characteristics, for evaluating subtle myocardial dysfunction [29], [30]. A close relationship between myocardial strain and long-term outcome in patients with myocardial dysfunction has been reported in a number of studies [31]. In addition, STE recognizes not only the different directions of myocardial strain but also the precise phase of the cardiac cycle [30]. To the best of our knowledge, STE has never been used to develop a mathematical model of the heart; thus, this is the first study that includes circumferential strain in a radial-directed thick-wall model. In contrast to the mathematical model, which does not consider strain, the mechanical model added the lower value of E caused by circumferential strain even while the positive relationship between E and 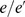 persisted. If the main purpose is to characterize the aging process of the human heart, the simpler cylinder model is adequate. Otherwise, more delicate factors should be considered (e.g., longitudinal strain).

The present study clearly showed that E was significantly higher in the elderly cohort than in the younger cohort, even under the same intraventricular pressure. E, which has never been used to describe the diastolic function using clinical data, has the potential to detect early diastolic dysfunction with high sensitivity. This will help us understand changes in myocardial stiffness with aging; therefore, we should be able to detect occult diastolic dysfunction in the early stage of systemic chronic diseases like hypertension disorder diabetes mellitus.

This study has several limitations. First, the main hypothesis was based on the linear correlation between the 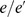 ratio and intraventricular pressure, but some confounding factors may interfere when measuring 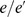 (e.g., tachycardia, frame rate, and different angles when sampling) [23]. Second, lacking sensitive circulating cardiac markers (e.g., troponin and brain natriuretic peptide), validating these formulas might underestimate overestimate asymptomatic heart failure [32], [33]. However, most of our patients were relatively healthy, according to the TOP study questionnaires [25]. Moreover, lifestyle-associated information was recorded for patients recruited from the TOP study but not for those given the health examination. Third, in patients without pericardial disease, extracardiac pressure was far lower than intraventricular pressure. One study [34] on 11 dogs with chronic heart failure reported that the pressure in the pericardial space was usually beneath 10 mmHg or 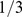 of the right atrial pressure. Thus, in most LV models, the extra cardiac pressure has been neglected. In our study, E in both models, whether or not extracardiac pressure was considered, showed a trend of changes similar to that of intraventricular pressure or IVSd. Fourth, we assumed the LV to be a hollow axisymmetric cylinder with plane strain, a state of strain in which the strain normal to the *x−y* plane, 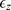, and the shear strain 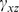 and 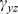, are assumed to be zero. The deformation in the axial direction (z direction) is ignored, and the strains in the radial and circumferential directions are independent of 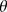. In reality, the left ventricle does not have a constant cross-section and does not deform uniformly at different orientations. In addition, the left ventricle was assumed to be made of elastic, isotropic, and homogeneous tissue when we determined the Young's modulus of the myocardium. Nonetheless, the left ventricle usually contains at least two basic raw materials, viz., muscle and collagen.

Although a mathematical model has limitations, it enables us to illustrate the quantitative relationships between structural and functional changes. because diastolic and systolic heart failure share similar poor outcomes [35] detecting diastolic dysfunction early helps to allow early interventions, which may lead to a better prognosis [36]. Furthermore, this model uses echocardiographic parameters instead of histological data, and it is noninvasive, it makes early detection more feasible.

In conclusion, this is the first report to document using a mathematical model to delineate diastolic dysfunction in the aging human heart. The vulnerability of the elderly to higher pressure may contribute to their developing earlier signals for heart failure. E is useful for the early and noninvasive detection of diastolic dysfunction.

## Supporting Information

Table S1 After excluding patients with hypertension, diabetes mellitus, and coronary artery disease, the correlation between E and diastolic dysfunction remained significant in the healthy elders.
(DOC)

Table S2 Multivariate analysis on the relationship between E and echocardiographic intraventricular pressure in younger patients.
(DOCX)

Table S3 Multivariate analysis on the relationship between E and echocardiographic intraventricular pressure in older patients.
(DOCX)
