# Supplementary material for: Characterization of Aging-Associated Cardiac Diastolic Dysfunction
Source: PLoS One. 2014 May 28;9(5):e97455. doi: 10.1371/journal.pone.0097455 (PMC4037178; doi:10.1371/journal.pone.0097455)
Supplement: Table S1 — After excluding patients with hypertension, diabetes mellitus, and coronary artery disease, the correlation between E and diastolic dysfunction remained significant in the healthy elders. (DOC) [file pone.0097455.s001.doc]

Supplementary Table S1. Comparison of E value, clinical, and echocardiographic parameters between older patients with preserved and impaired diastolic function in the subgroups of healthy elderly, elderly with hypertension, elderly with DM, elderly with CAD

|  | Healthy elderly | | Elderly with HTN | | Elderly with DM | | Elderly with CAD | |
| --- | --- | --- | --- | --- | --- | --- | --- | --- |
|  | n = 167 (23.55%) | | n = 349 (49.22%) | | n = 122 (17.20%) | | n = 71 (10.01%) | |
|  | Normal |  | Normal |  | Normal |  | Normal |  |
|  | diastolic | Diastolic | diastolic | Diastolic | diastolic | Diastolic | diastolic | Diastolic |
|  | function | dysfunction | function | dysfunction | function | dysfunction | function | dysfunction |
|  | n = 141 | n = 26 | n = 209 | n = 140 | n = 67 | n = 55 | n = 29 | n = 42 |
| Variable | (84.40%) | (15.50%) | (59.88%) | (40.11%) | (54.90%) | (45.00%) | (40.80 %) | (59.10%) |
| Age (years) | 67.81 ± 19.83 | 79.81 ± | 71.28 ± | 72.31 ± 9.8 | 69.71 ± | 71.32 ± | 70.43 ± | 68.21 ± 8.3 |
|  |  | 11.85* | 17.35 |  | 14.31 | 21.34 | 17.42 |  |
| Male | 112 (79.4) | 6 (23.07)* | 127 (60.76) | 59 (42.14) | 36 (53.73) | 20 (36.4) | 20 (68.9) | 27 (64.2) |
| IVSD (cm) | 0.73 ± 0.21 | 0.91 ± 0.3 | 1.01 ± 0.1 | 1.21 ± 0.24 | 0.93 ± 0.31 | 1.09 ± 0.25 | 0.65 ± 0.17 | 1.13 ± 0.2 |
| LVPWd (cm) | 0.68 ± 0.37 | 1.09 ± 0.4 | 1.07 ± 0.31 | 1.12 ± 0.6 | 0.74 ± 0.57 | 0.87 ± 0.48 | 0.6 ± 0.3 | 0.89 ± 0.4 |
| LVIDd (cm) | 3.78 ± 0.5 | 5.43 ± 0.43 | 4.51 ± 1.1 | 4.09 ± 0.87 | 4.5 ± 0.65 | 4.3 ± 0.7 | 4.67 ± 0.8 | 5.2 ± 0.64 |
| LVIDs (cm) | 3.26 ± 0.71 | 2.8 ± 0.4 | 2.55 ± 0.38 | 2.17 ± 0.7 | 2.66 ± 0.6 | 2.79 ± 0.58 | 2.77 ± 0.71 | 3.18 ± 0.9 |
| LVEF (%) | 74.36 ± 8.2 | 77.65 ± 7.1 | 64.2 ± 6.8 | 62.64 ± 7.2 | 72.95 ± 6.4 | 67.98 ± 5.8 | 71.99 ± 7.3 | 68.79 ± 5.7 |
| e (m/s) | 0.78 ± 0.37 | 0.54 ± 0.29 | 0.87 ± 0.12 | 1 ± 0.41 | 0.72 ± 0.21 | 0.58 ± 0.15 | 0.45 ± 0.2 | 0.57 ± 0.18 |
| e/a | 0.98 ± 0.09 | 0.67 ± 0.41 | 0.89 ± 0.34 | 0.71 ± 0.21 | 0.93 ± 0.2 | 0.64 ± 0.31 | 0.91 ± 0.42 | 0.58 ± 0.11 |
| e′ (m/s) | 0.1 ± 0.01 | 0.06 ± 0.02* | 0.09 ± 0.01 | 0.07 ± 0.03 | 0.1 ± 0.02 | 0.06 ± 0.01* | 0.08 ± 0.03 | 0.05 ± 0.02 |
| e/e′ | 7.8 ± 2.1 | 10.8 ± 4* | 9.06 ± 3.8 | 10.4 ± 2.7 | 7.2 ± 4.3 | 9.66 ± 4.1 | 5.62 ± 3.8 | 11.4 ± 3.1* |
| IVRT | 121 ± 31.2 | 95.3 ± 24.3 | 102.7 ± 21.2 | 93.1 ± 19.8 | 116.5 ± 23.1 | 85 ± 17.3 | 135.8 ± 19.8 | 76.3 ± 24.31* |
| DT | 167.7 ± 58.3 | 200.1 ± 68.3 | 175.5 ± 38.4 | 205 ± 47.1 | 166.2 ± 48.2 | 207.4 ± 59.1 | 216.8 ± 68.1 | 201.1 ± 49.3 |
| E | 25808.1 ± 7102.4 | 45637.34 ± | 39852.85 ± | 40385.4 ± | 29835.31 ± | 35637.75 ± | 27354.14 ± | 41589 ± |
| (Young’s |  | 6852.1* | 4321.4 | 3812.1 | 8423.1 | 10452.1* | 7431.2* | 7234.5 |
| modulus) |  |  |  |  |  |  |  |  |

HTN = hypertension; DM = diabetes mellitus; CAD = coronary artery disease; IVSd = inter-ventricular septal diameter in diastolic phase; LVPWd = left ventricular posterior wall diameter in diastolic phase; LVIDd = left ventricular internal diastolic dimension; LVIDs = left ventricular internal systolic dimension; e = early diastolic mitral inflow velocity; e/a = the ratio of early to late diastolic mitral inflow velocity; e′ = the average early diastolic velocity of mitral annulus in tissue Doppler; LVEF = left ventricular ejection fraction

*p < 0.05.
