# Supplementary material for: Characterization of Aging-Associated Cardiac Diastolic Dysfunction
Source: PLoS One. 2014 May 28;9(5):e97455. doi: 10.1371/journal.pone.0097455 (PMC4037178; doi:10.1371/journal.pone.0097455)
Supplement: Table S2 — Multivariate analysis on the relationship between E and echocardiographic intraventricular pressure in younger patients. (DOCX) [file pone.0097455.s002.docx]

Supplemental Table S2. The clinical and echocardiographic characteristics of younger populations with preserved or impaired diastolic function

|  | Normal diastolic function (e' ≥ 0.08) | Diastolic dysfunction (e' < 0.08) |  |
| --- | --- | --- | --- |
| Variable | n = 164 (77.72%) | n = 47 (22.27%) | p-value |
| Age (years) | 44.22 ± 4.92 | 54.31 ± 8.31 | 0.040 |
| Male | 109 (66.46) | 22 (46.8) | 0.040 |
| HTN | 21 (12.8) | 4 (8.51) | 0.210 |
| DM | 7 (4.26) | 4 (8.51) | 0. 010 |
| CAD | 4 (2.43) | 2 (4.25) | 0.070 |
| Echocardiographic parameters | | | |
| IVSd (cm) | 0.78 ± 0.21 | 0.93 ± 0.28 | 0.050 |
| LVPWd (cm) | 0.74 ± 0.10 | 0.79 ± 0.21 | 0.470 |
| LVIDd (cm) | 4.67 ± 0.45 | 4.92 ± 0.47 | 0.950 |
| LVIDs (cm) | 2.78 ± 0.4 | 2.89 ± 0.38 | 0.840 |
| LVEF (%) | 69.21 ± 8.10 | 69.49 ± 5.20 | 0.900 |
| e (m/s) | 0.73 ± 0.29 | 0.69 ± 0.12 | 0.650 |
| e/a | 1.12 ± 0.63 | 0.72 ± 0.42 | 0.620 |
| e′ (m/s) | 0.1 ± 0.03 | 0.06 ± 0.01 | 0.001 |
| e/e′ | 6.09 ± 1.13 | 10.04 ± 4.28 | 0.001 |
| IVRT | 103.42 ± 20.97 | 84.96 ± 17.49 | 0.006 |
| DT | 198.88 ± 75.81 | 222.31 ± 6.90 | 0.31 |
| E (Young’s modulus) | 27031.15 ± 8431.20 | 34281.06 ± 9412.30 | 0.007 |

Data are means ± SD. Abbreviations: see Supplementary Table 1.
