# Supplementary material for: Characterization of Aging-Associated Cardiac Diastolic Dysfunction
Source: PLoS One. 2014 May 28;9(5):e97455. doi: 10.1371/journal.pone.0097455 (PMC4037178; doi:10.1371/journal.pone.0097455)
Supplement: Table S3 — Multivariate analysis on the relationship between E and echocardiographic intraventricular pressure in older patients. (DOCX) [file pone.0097455.s003.docx]

Supplemental Table S3. The clinical and echocardiographic characteristics of older populations with preserved or impaired diastolic function

|  | Normal diastolic function (e' ≥ 0.08) | Diastolic dysfunction (e' < 0.08) |  |
| --- | --- | --- | --- |
| Variable | n = 460 (64.88 %) | n = 249 (35.11%) | p-value |
| Age (years) | 72.89 ± 6.53 | 74.16 ± 7.81 | 0.92 |
| Male | 376 (81.73) | 108 (43.37) | 0.4 |
| HTN | 224 (48.69) | 125 (50.2) | 0.82 |
| DM | 47 (16.08) | 48 (19.27) | 0. 1 |
| CAD | 33 (7.17) | 38 (15.26) | 0.05 |
| Echocardiographic parameters | | | |
| IVSd (cm) | 0.76 ± 0.22 | 0.89 ± 0.41 | 0.1 |
| LVPWd (cm) | 1.18 ± 5.60 | 1.22 ± 7.12 | 0.74 |
| LVIDd (cm) | 4.98 ± 0.48 | 5.21 ± 0.71 | 0.45 |
| LVIDs (cm) | 3.01 ± 0.25 | 3.12 ± 0.64 | 0.78 |
| LVEF (%) | 67.64 ± 8.91 | 68.00 ± 7.00 | 0.95 |
| e (m/s) | 0.65 ± 0.17 | 0.64 ± 0.17 | 0.92 |
| e/a | 0.91 ± 0.27 | 0.65 ± 0.09 | 0.05 |
| e′ (m/s) | 0.09 ± 0.03 | 0.05 ± 0.01 | 0.01 |
| e/e′ | 7.57 ± 2.25 | 10.38 ± 3.29 | 0.02 |
| IVRT | 114.83 ± 18.17 | 93.66 ± 13.65 | 0.12 |
| DT | 181.66 ± 86.27 | 233.66 ± 120.55 | 0.47 |
| E (Young’s modulus) | 27931.94 ± 4534.12 | 39876.37 ± 9731.23 | 0.001 |

Data are means ± SD. Abbreviations: see Supplementary Table 1.
